# Osseous defect of the anteroinferior femoral head: is it associated with femoroacetabular impingement (FAI)?

**DOI:** 10.1007/s00256-021-03730-x

**Published:** 2021-02-04

**Authors:** Franca K. Boldt, Benjamin Fritz, Patrick O. Zingg, Reto Sutter, Christian W. A. Pfirrmann

**Affiliations:** Balgrist University Hospital, University of Zurich, Forchstrasse 340, 8008 Zürich, Switzerland

**Keywords:** Hip arthrography, Magnetic resonance imaging, Femoral acetabular impingement

## Abstract

**Objective:**

To evaluate the prevalence, morphology, and clinical significance of a repeatedly observed yet not examined circumscript osseous defect at the anteroinferior aspect of the femoral head, termed femoral head defect.

**Materials and methods:**

Retrospective study with approval of the institutional review board. There was informed consent by all individuals. Magnetic resonance imaging (MRI) hip examinations of 970 individuals (age 15 to 55) were analyzed for femoral head defect. Patients with femoral head defect were matched for age and gender with patients without defect. Two readers independently assessed MRI images regarding presence, location, and morphology of the defect. MR images and radiographs were analyzed for findings of femoroacetabular impingement (FAI). Femoral torsion was measured. Independent *t* test and chi-square test were used for statistics.

**Results:**

Sixty-eight (7%) of 970 MRI examinations exhibited a femoral head defect in an anteroinferior location of the femoral head (29/400 men, 7.3%; 39/570 women, 6.8%; *p =* 0.8). The most frequent morphology of femoral head defect was type I, dent-like (34; 50%), followed by type II, crater-like (27; 40%), and III, cystic (7; 10%). Femoral head defect was slightly more common on the right hip (39 individuals; 57%) compared to left (29 individuals; 43%), non-significantly (*p =* 0.115). There was no association between FAI or its subtypes and the presence of femoral head defect (*p =* 0.890). Femoral antetorsion was reduced in patients with femoral head defect (12.9° ± 8.6) compared to patients without defect (15.2° ± 8.5), without statistical significance (*p =* 0.121).

**Conclusion:**

The femoral head defect is a common finding in MRI examinations of the hip and is situated in the anteroinferior location. There was no association with FAI yet a non-significant trend towards lower femoral antetorsion in patients with femoral head defects.

## Introduction

A subchondral osseous defect of the anteroinferior femoral head, termed femoral head defect (FHD), has been repeatedly observed in our clinical practice. The location of this finding is distinct from the location of the widely described herniation pit located at the anterosuperior femoral head [1–3]. To date, there are only two studies mentioning osseous defects of the anteroinferior femoral head oversimplifying the finding as a mere variant of the herniation pit [4, 5]. There has been no in-depth analysis of the FHD with exploration of possible associations with femoroacetabular impingement or abnormal femoral antetorsion. The missing data on FHD could result in diagnostic pitfalls; i.e., this finding could be mistaken for osteonecrosis, fracture, or a subchondral cyst. Therefore, we set out to describe the morphological characteristics and prevalence of the FHD in patients who received MR arthrography of the hip joint.

## Material and methods

### Study population

This retrospective study was approved by the institutional review board. All individuals granted informed consent.

All patients with MR arthrography of the hip and conventional radiographs of the pelvis and hip were included between January 2016 and March 2018: 970 patients between the ages of 15 and 55 years (mean age 34 years), 400 men and 570 women, were included in this study. All patients were referred for MR arthrography by specialized hip surgeons because of pain in the hip or groin. Forty-two percent of patients having been referred for MRI were previously diagnosed with FAI. FAI was clinically suspected in 24%. Thirty-two percent of patients were referred with unclear hip pain without trauma, and 2% with pain after trauma. All MR arthrographies of the hip and all radiographic studies were performed by the radiology department of our university hospital. Exclusion criteria consisted of advanced osteoarthritis of the hip corresponding to grade 3 or 4 according to the Kellgren and Lawrence classification, systemic rheumatological disease, neoplasm of the hip, pigmented villonodular synovitis (PVNS), prior surgery of the hip, hip dysplasia, Perthes disease, as well as coxa magna.

### MR imaging

All patients referred to our department underwent MR arthrography in the following standardized manner. A musculoskeletal radiologist performed a fluoroscopy-guided intraarticular injection of first 1 mL of local anesthetic (lidocaine hydrochloride 2%, Rapidocain; Sintetica, Mendrisio, Switzerland) under aseptic conditions followed by 1 mL of iodinated contrast agent (iopamidol 200 mg/mL, Iopamiro 200; Bracco, Milan, Italy). Having verified the intraarticular distribution of the contrast agent, the radiologist subsequently injected 15–20 mL of MR contrast medium gadopentetate dimeglumine 2 mmol/L (Magnevist, Bayer Healthcare, Berlin, Germany). This allows for a good distention of the hip joint and optimal examination of the labrum and cartilage.

The patients were then taken to the MRI scanner, the elapsed time between the injection and MR image acquisition not surpassing 15 min. As the fluoroscopy room and the MRI are in close proximity, the patients usually walk the short distance. If there is a short waiting time between the injection and the MRI, the patients lie down on a gurney.

MR images were acquired on a 1.5 T system (Avanto fit, Siemens Healthcare, Erlangen, Germany). For hip imaging, a body matrix surface coil was placed over the hip of the supine patient combined with a spine matrix coil integrated in the MRI table. The routine MRI protocol employed by our institution (Table 1) consists of first a three-dimensional data set with a transverse oblique, paralleling the femoral neck axis water-excitation true fast imaging with steady-state precession gradient-echo sequence (FISP). This data set was used for reformatting radial images perpendicular to the short axis of the femoral neck. Next followed the acquisition of a coronal T1-weighted spin-echo (SE) sequence and a coronal intermediate-balanced fast spin-echo sequence with fat saturation. Sagittal water-excitation three-dimensional double-echo steady-state sequence was acquired. To determine the femoral torsion, the following short sequences were acquired: A transverse T2-weighted fast spin-echo sequence over the femoral head and neck followed by a transverse T2-weighted sequence was performed over the femoral condyles.

**Table 1:** Routine protocol for MR arthrography of the hip

Parameter	Coronal T1-weighted TSE	Coronal intermediate-weighted FS TSE	Oblique transverse True FISP	Sagittal True FISP	Transverse T2-weighted HASTE: Hip	Transverse T2-weighted HASTE: Knee
Repetition time (msec)/echo time (msec)	600/13	2500/25	10.76/4.66	25.01/8.56	1000/93	1400/93
Section thickness (mm)	3	3	1	1.7	5	5
Field of view (mm)	180 × 180	180 × 180	170 × 170	159 × 159	240 × 240	240 × 240
Matrix	269 × 384	320 × 320	269 × 384	269 × 384	256 × 256	256 × 256
Echo train length	3	7	1	2	126	154
Pixel bandwidth (Hz/pixel)	130	130	200	130	700	700
No. of signals acquired	2	1	1	1	1	1
Acquisition time (min:sec)	3:39	3:57	4:15	4:22	0:25	0:14

*FS* fat saturated, *TSE* turbo spin-echo, *FISP* true fast imaging with steady-state precession, *HASTE* half-Fourier acquisition single-shot turbo spin-echo

### Radiographs of the pelvis and hip

Radiographs of the pelvis and hip were routinely obtained in patients with groin pain in anteroposterior projection of the pelvis as well as a cross-table lateral view of the hip. To ensure an accurate pelvic tilt on the anteroposterior radiograph of the pelvis, special attention was given to the craniocaudal distance of sacrococcygeal joint to the pubic symphysis (average about 3.2 cm in men and 4.7 cm in women) [6].

### Image analysis

#### Prevalence and location of the femoral head defect

**Fig. 1:**
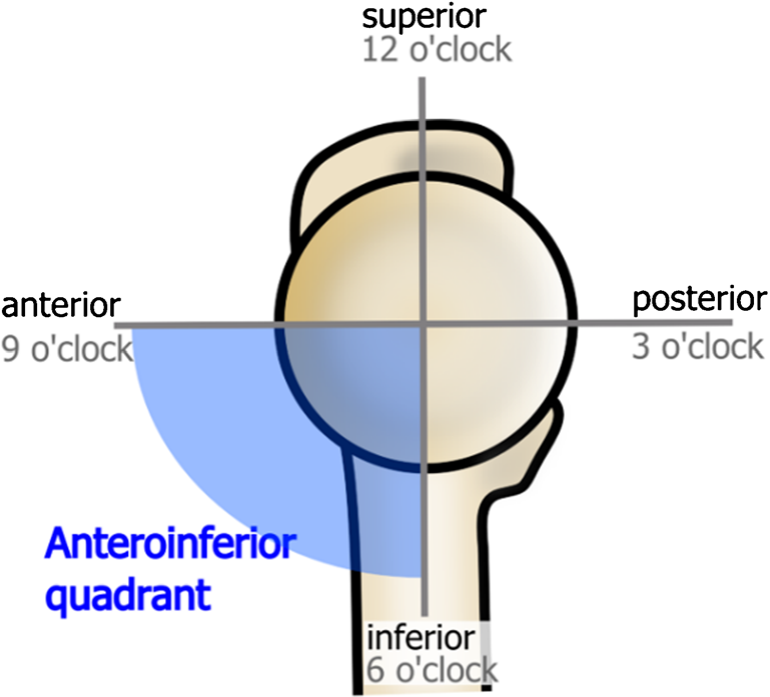
Schematic drawing of the medial view of the femoral head and proximal femur indicating the spatial orientation and schematic clockface used to localize the FHD in the anteroinferior quadrant

All MRI examinations were evaluated for the presence of an osseous defect in the anteroinferior aspect of the femoral head, termed femoral head defect (FHD). All 970 MRI examinations were independently assessed by two fellowship-trained musculoskeletal radiologists for the presence and location of FHD and categorized its morphology. Both radiologists were blinded for review. The location of the FHD was determined in a sagittal plane using a schematic clock face, 9:00 being the anterior position on a hypothetical horizontal line through the middle of the femoral head, 3:00 posterior position, 12:00 defined as the superior position on a vertical line through the center of the femoral head, and 6:00 as the inferior position the same line (Fig. 1). Only defects in the anteroinferior quadrant of the femoral head were recorded as FHD. Lesions in the anterosuperior, superior, and posterosuperior location of the femoral head were counted as herniation pits.

**Fig. 2:**
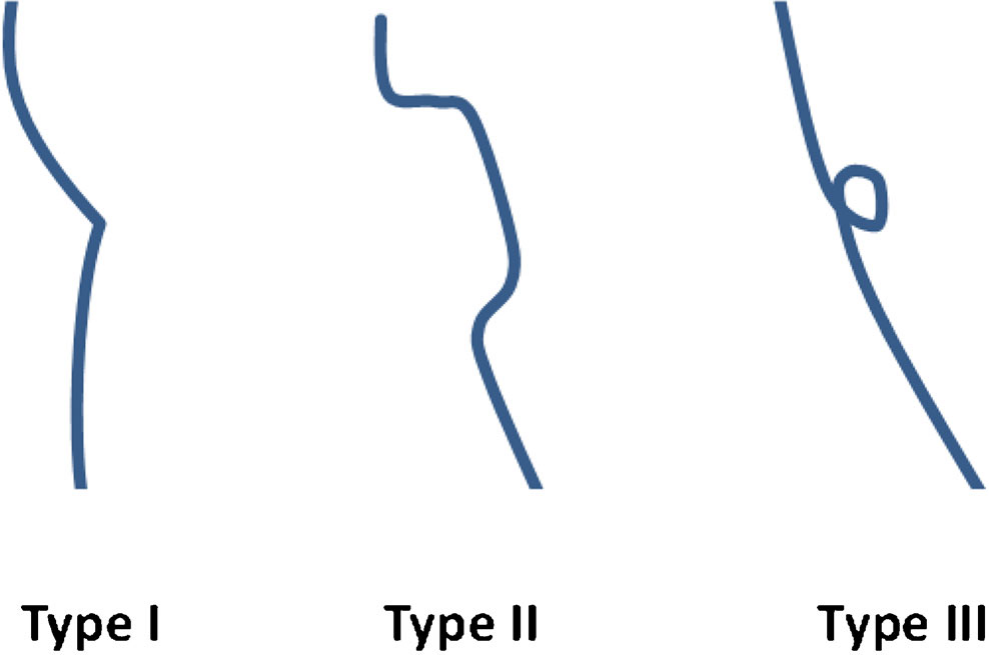
Morphological types of femoral head defect (FHD) shown in a schematic drawing. Type I: pointed, dent-like defect. Type II: crater-like depression. Type III: round, cyst-like defect

**Fig. 3:**
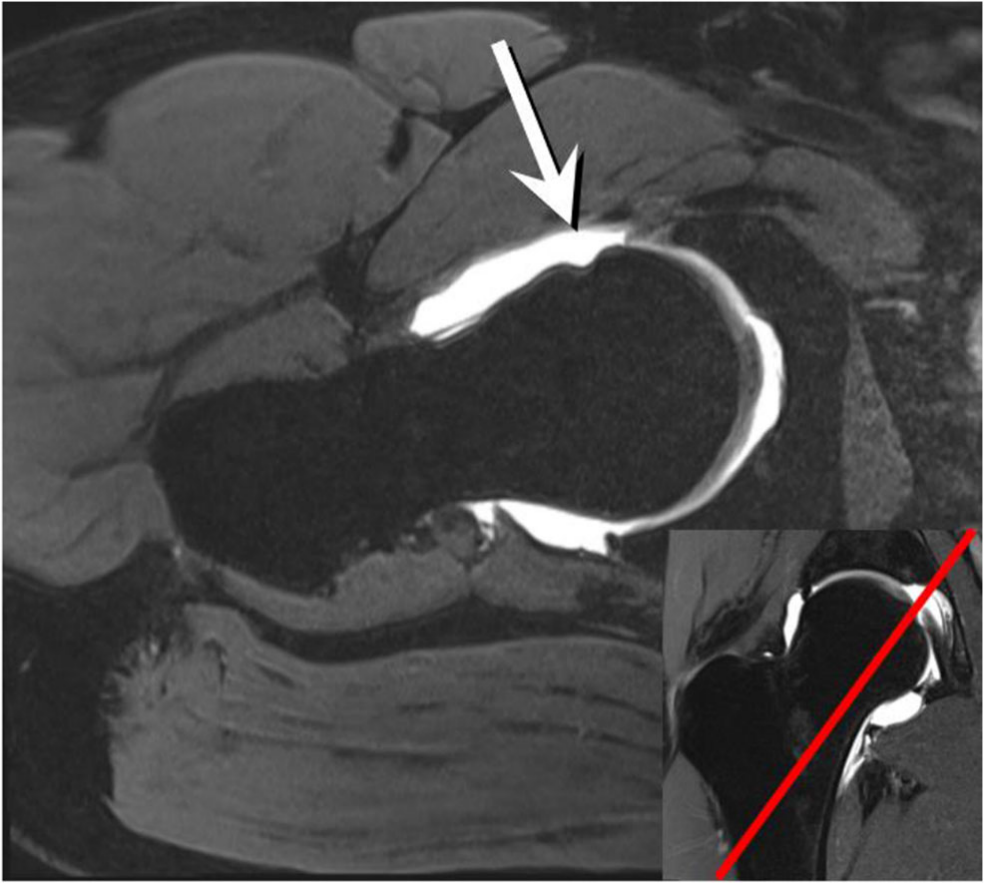
Transverse oblique three-dimensional water-excitation true fast imaging with steady-state precession MR arthrography image (repetition time msec/echo time msec, 12/6) of the right hip of a 35-year-old man. Osseous dent-like defect at the anteroinferior femoral head (arrow) corresponding to a type I femoral head defect (FHD). Type I FHD at the anteroinferior femoral head (arrow). Insert Coronal image with a red reference line indicating the location of the transverse oblique image, confirming the location of the FHD in an anteroinferior location

**Fig. 4:**
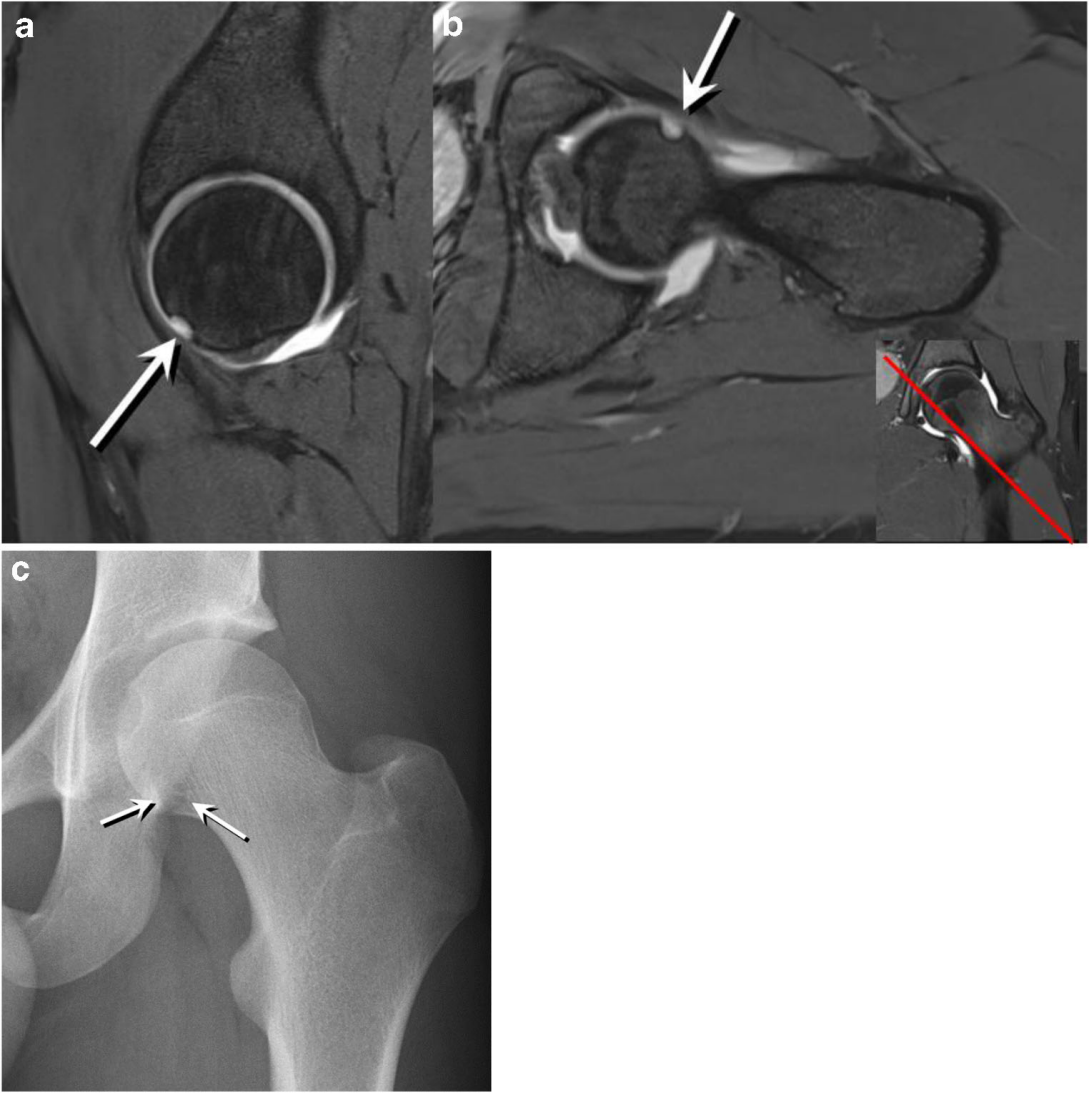
**a** Sagittal 3D double-echo steady-state MR arthrography image (25/9) of the left hip of an 18-year-old man. Osseous crater-like defect at the anteroinferior femoral head (arrow) corresponding to a type II FHD. **b** Transverse oblique three-dimensional water-excitation true fast imaging with steady-state precession MR arthrography image (repetition time msec/echo time msec, 12/6) of the left hip of the same patient. Type II FHD at the anteroinferior femoral head (arrow). Insert in **b** Coronal image with a red reference line indicating the location of the transverse oblique image, confirming the location of the FHD in an anteroinferior location. **c** AP pelvic radiograph of the same patient. Crater-like type II FHD at the anteroinferior femoral head (arrows)

**Fig. 5:**
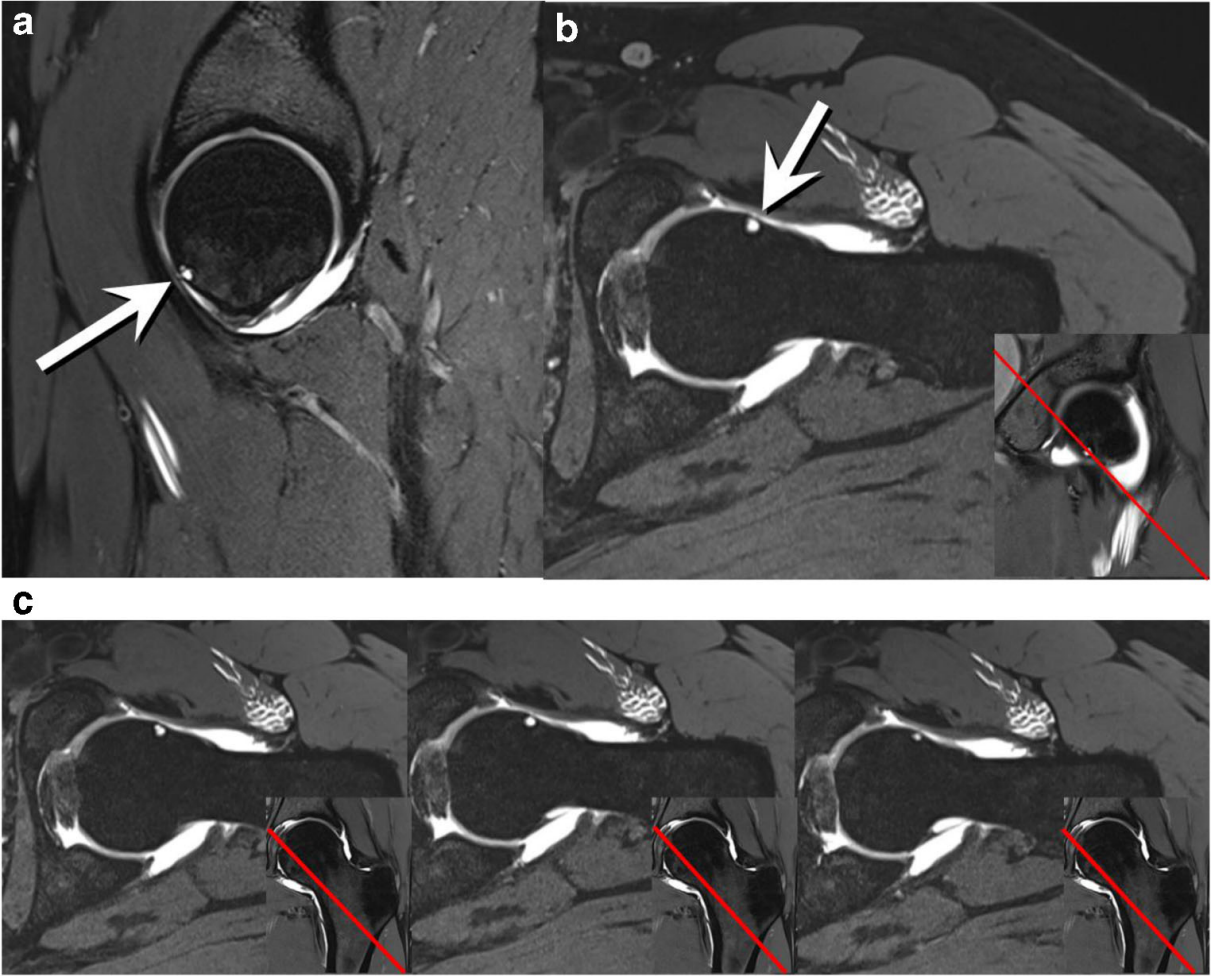
**a** Sagittal 3D double-echo steady-state MR arthrography image (25/9) of the left hip of a 31-year-old woman. Round, cyst-like subcortical defect (arrow) corresponding to a type III femoral head defect (FHD). **b** Transverse oblique three-dimensional water-excitation true fast imaging with steady-state precession MR arthrography image (repetition time msec/echo time msec, 12/6) of the left hip of the same patient. Type III FHD at the anteroinferior femoral head (arrow). Insert in **b** Coronal image with a red reference line indicating the location of the transverse oblique image, confirming the location of the FHD in an anteroinferior location. **c** Series of oblique images from the same sequence described in **b** from superior to inferior, with inserts indicating the position of the respective images on coronal images with a red reference line, confirming the location of the FHD in an anteroinferior location

Based on the data seen in our study, the configuration of FHD on sagittal MRI planes appeared in three main different morphologies, and we therefore propose a simple classification system in our study to address morphology. FHD were subdivided in morphological subgroups based on their appearance (Fig. 2): Type I was defined as a pointed, dent-like osseous defect of the anteroinferior femoral head (Fig. 3). Type II represented a groove or crater-like depression (Fig. 4). Cystic subcortical lesion with a minimal cortical opening was termed type III (Fig. 5).

#### Analysis of the femoral head defect

In a sub-analysis, each patient exhibiting a FHD was matched with a patient of the same sex and age not displaying a femoral defect, resulting in 68 patients with FHD and a matched group of 68 patients without FHD. For this sub-analysis, a fellowship-trained musculoskeletal radiologist who was blinded for review performed additional measurements on the MRI examinations and the radiographic studies. To avoid mistaking the observed defect for an epiphyseal remnant, the distance between the fused epiphyseal plate and the FHD was measured in the coronal plane with the FHD located laterally to the fused epiphyseal plate.

The femoral torsion, an important factor for the development of FAI, was measured on axial MRI images. According to the clinically employed method described by Tomczak [7], the femoral torsion constitutes the angle between the longitudinal axis of the femoral neck and the tangent posterior to the distal femur condyles. A normal femoral antetorsion was defined as 13° ± 8°, a pathological femoral torsion being lower than 3° or higher than 23° [8].

MRI images were analyzed for the cam-type morphology defined as a non-spherical femoral head-neck junction and graded on radial sequences according to the score established by Reichenbach [9]. Grades from 0 to 3 were ascribed as 0: normal, no evidence of a non-spherical femoral shape on any of the sequences; 1: possible deformity with cortical irregularity and a possible mild decrease of the anterior head-neck offset; 2: definite deformity with an established decrease of the anterior head-neck offset; 3: severe deformity with a large decrease of the anterior head-neck offset.

The anteroposterior pelvic radiographs were examined for signs of pincer-type femoroacetabular impingement (FAI). In pincer-type FAI, there is a mechanical conflict of the acetabulum and femur due to general or focal acetabular overcoverage. The general acetabular coverage of the femoral head was determined by measuring the lateral center-edge angle (LCE angle; Wiberg angle), a normal range being 23–33°. LCE angles greater than 39° were defined as severe general overcoverage of the femoral head by the acetabulum. Focal overcoverage of the anterolateral hip joint may occur due to acetabular retroversion. Radiographic indicators of acetabular retroversion consist of following three signs. The crossover sign describes the anterior acetabular wall crossing the posterior wall. The ischial spine sign consists of the ischial spine protruding medially beyond the pelvic rim. The posterior wall sign is positive when the center of the femoral head lies laterally to the posterior acetabular wall [10, 11]. Pincer-type FAI was defined as a general acetabular overcoverage or at least two signs of acetabular retroversion being positive [11–14]. Patients with stand-alone pincer- or cam-type deformities were ascribed to the according subgroup. Patients with signs of both pincer- and cam-type FAI were categorized as mixed-type FAI.

As further assessment of the hip anatomy, the angle between the femoral neck and shaft, caput-collum-diaphyseal (CCD) angle, was measured. Normal values are considered from 120 to 135°. CCD angles greater than 135° constitute coxa valga, and angles lower than 120° coxa vara [15]. The valgus hip is known to be associated with a reduced internal rotation of the hip [16].

### Statistical analysis

The two-tailed Mann-Whitney *U* test was used to assess the difference in age and femoral torsion between the group of patients with and without FHD. *p* values of less than .05 were taken as proof of a significant difference.

The unpaired *t* test was used to differentiate the femoral torsion of patients with and without FHD according to gender. The chi-square test was employed to determine the number of male and female patients in each group as well the number of patients with FHD in each FAI subcategory. Descriptive statistics were utilized in describing the morphology and dimension of FHD, location of FHD, femoral antetorsion of the subgroups; mean values, as well as standard deviations, were calculated. The Cohen kappa for categorical data was used to determine the interobserver agreement. Kappa values of 0.61 to 0.8 were taken to indicate substantial to good interreader agreement. Kappa values greater than 0.8 meant very good to almost perfect agreement. The statistic software utilized in this study was SPSS for Windows, 17.0; SPSS, Chicago, III.

## Results

### Demographics

Sixty-eight (7%) of 970 MRI examinations exhibited FHD in an anteroinferior location of the femoral head (29/400 men, 7.3%; 39/570 women, 6.8%; *p =* 0.8).

There was no significant difference between the mean age of patients with FHD (29.3 years ± 10.8) and those without FHD (29.3 years ± 10.7; *p* = 0.8). Men with FHD were not significantly younger (29.9 years ± 10), than women with FHD (31.3 years ± 11; *p* = 0.580). FHD was slightly more common on the right hip (39/68 individuals; 57%) compared to the left (29/68 individuals; 43%), which was not statistically significant (*p =* 0.115).

### Morphology of the FHD

**Fig. 6:**
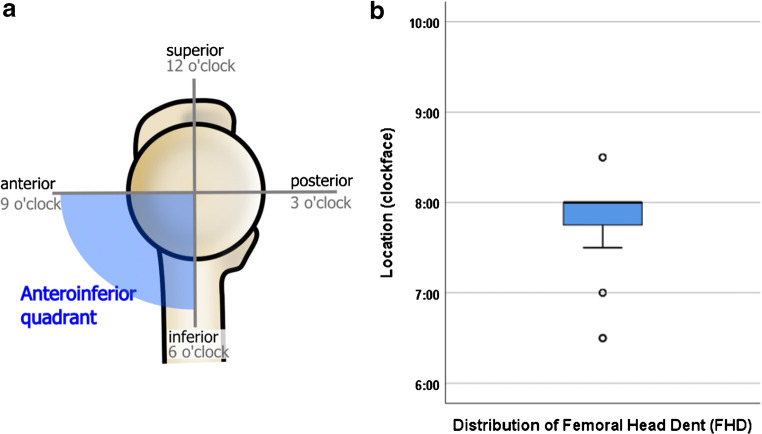
**a** Schematic drawing of the medial view of the femoral head and proximal femur indicating the spatial orientation and schematic clockface used to localize the FHD in the anteroinferior quadrant. **b** Boxplot showing the distribution of FHD according to location on the schematic clockface between the 6:30 and 8:30 position. Solid box includes first to third quartile; the dark line at the top of the box is the median (the overwhelming majority of FHD were located at the 8 o’clock position). Whisker indicates 95% confidence interval, and dots indicate outliers

The most frequent morphology of FHD was type I (34/68; 50%), followed by type II (27/68; 40%) and III (7/68; 10%). The mean distance of FHD to the scar of the physis was 9.9 mm ± 3.7 (min. 2.3 mm, max. 18 mm) measured on a coronal plane confirming that the FHD is not a physeal remnant. On a clockface of the femoral head on a sagittal plane, the defect was most often located at the 8 o’clock position (± 0.4, range from 6:30 to 8:30) (Fig. 6).

The mean height of the defect measured on the sagittal plane was 3.4 mm ± 1.7, the length on sagittal plane 2 mm ± 1.1, and the mean width in axial plane 3.4 mm ± 1.3.

Bone marrow edema surrounding the FHD was present in 3% (2/68 patients). Directly adjacent to the FHD, no articular cartilage was seen. Nine of 68 (13%) patients with FHD also showed a herniation pit. Thirteen of 68 (19%) of the matched patients without a FHD had herniation pits.

### Interobserver variability

The interobserver agreement for the presence of FHD was 0.909 ± 0.045.

The interobserver agreement for determining the morphological category of FHD was 0.933 ± 0.066. Both readers thus showed almost perfect agreement.

### Femoroacetabular impingement

The diagnosis of femoroacetabular impingement (FAI) was established by correlation with the clinical findings of an orthopedic specialist. There was no association between FAI or its subtypes and the presence of an FHD (*p =* 0.890). Thirty-five percent of patients with FHD showed a pincer-type FAI (24/68), 13% cam-type (9/68), 41% mixed-type FAI (28/68); 10% showed no signs of FAI (7/68).

Thirty-four percent of the matched study group showed a pincer-type FAI (23/68), 12% cam-type FAI (8/68), and 40% combined FAI (27/68); 14% of patients had no signs of FAI (10/68).

In patients with no features of FAI, other pathologies explaining the hip symptoms were observed: borderline dysplasia (3%), synovial chondromatosis (1%), subspine impingement following avulsion fracture of the anterior inferior iliac spine (1%), insufficiency fracture of the femoral head (2%), and coxa valga (2%). Some patients with FAI had coexisting pathologies contributing to their hip pain: avulsion fracture of the anterior inferior iliac spine (1%), femoral neck insufficiency fracture (1%), tendinopathy of hip abductors (2%), ischiofemoral impingement (1%).

### Femoral antetorsion

Femoral antetorsion was reduced in patients with FHD (12.9° ± 8.6) compared to patients without FHD (15.2° ± 8.5), although without statistical significance (*p =* 0.121).

Women with FHD had a more pronounced lower femoral torsion (13.5° ± 8.85) than women without FHD (16.3° ± 8.6), though without statistical significance (*p =* 0.155). Women with FHD showed a slightly lower femoral torsion (13.5° ± 8.9) vs. men with FHD (12.1 ± 8.4) *p =* 0.155).

Also, women with abnormally low femoral antetorsion (< 3°) showed a higher prevalence of FHD (5.7%) than women without FHD (2.3%), without statistical significance (*p =* 0.411).

Women with abnormally high femoral antetorsion (> 23°) less often had a FHD (10.15% vs. 4.6%) (*p =* 0.549).

### LCE angle and CCD angle

Patients with and without FHD showed no significant difference in LCE angles (patients with FHD 32.6° ± 6.2, without FHD 34.2° ± 6.4; *p =* 0.142). Patients with and without FHD showed no significant difference in CCD angles (patients with FHD 132.3° ± 5.5, without FHD 132.7° ± 5; *p* = 0.629).

### Appearance of FHD in arthroscopy

**Fig. 7:**
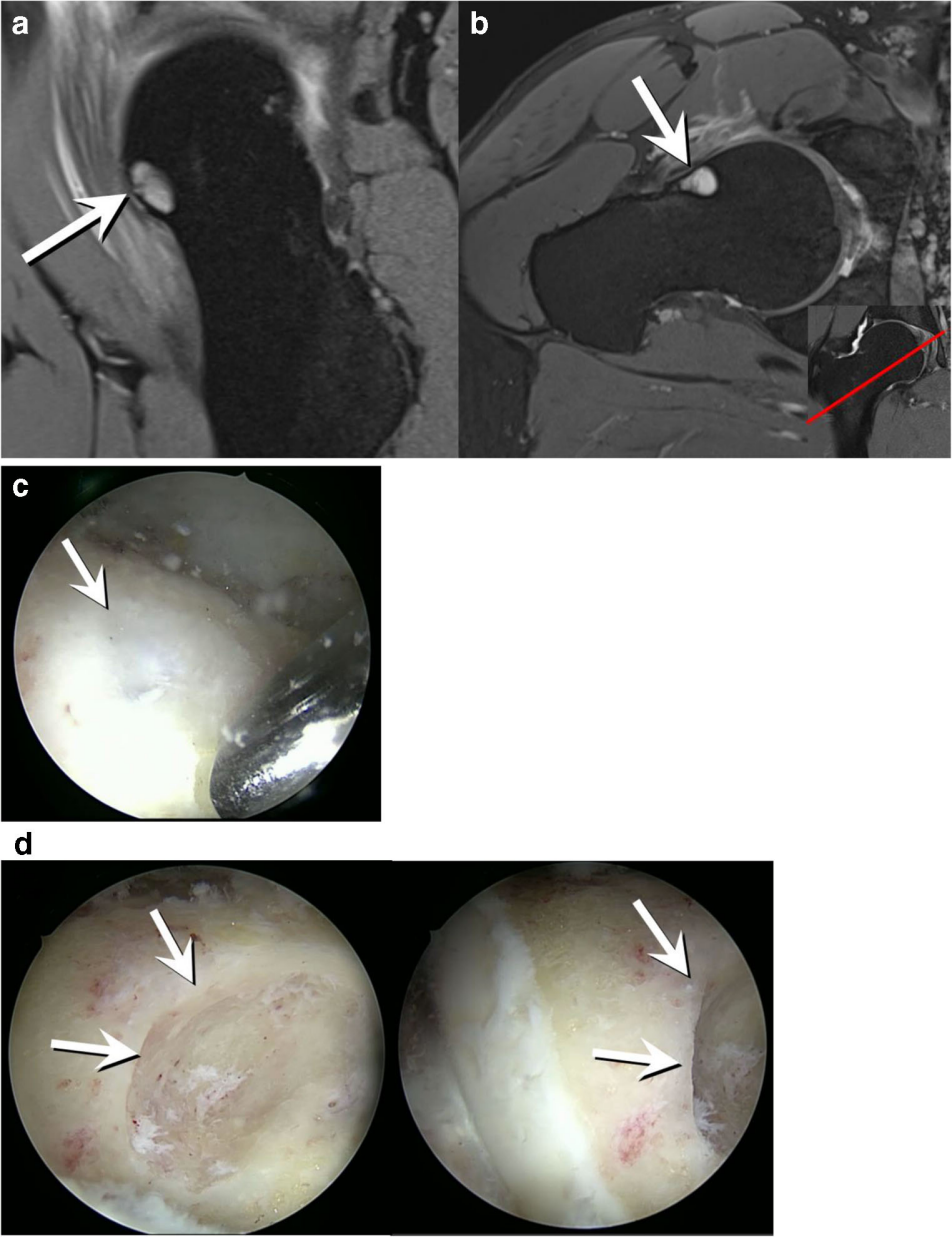
**a** Preoperative sagittal 3D double-echo steady-state MR arthrography image (repetition time msec/echo time msec 25/9) of the right hip of a 40-year-old man with mixed-type FAI. Round, cystic subcortical defect (arrow) corresponding to a type III FHD. **b** Transverse oblique three-dimensional water-excitation true fast imaging with steady-state precession MR arthrography image (repetition time msec/echo time msec, 12/6). Type III FHD at the anteroinferior femoral head (arrow). Insert in **b** Coronal image with a red reference line indicating the location of the transverse oblique image, confirming the location of the FHD in an anteroinferior location. **c** Arthroscopic intraoperative appearance of the FHD (arrow). The patient was arthroscopically treated for FAI by resection of the cam deformity (osteochondroplasty) as well as refixation of a labral tear (not depicted here). The initial arthroscopic picture at the beginning of the procedure shows a shallow surface alteration of the femoral head covering the FHD (arrow). **d** After arthroscopic deroofing, a crater-like sclerotic base of the cystic FHD (arrows) is visible

**Fig. 8:**
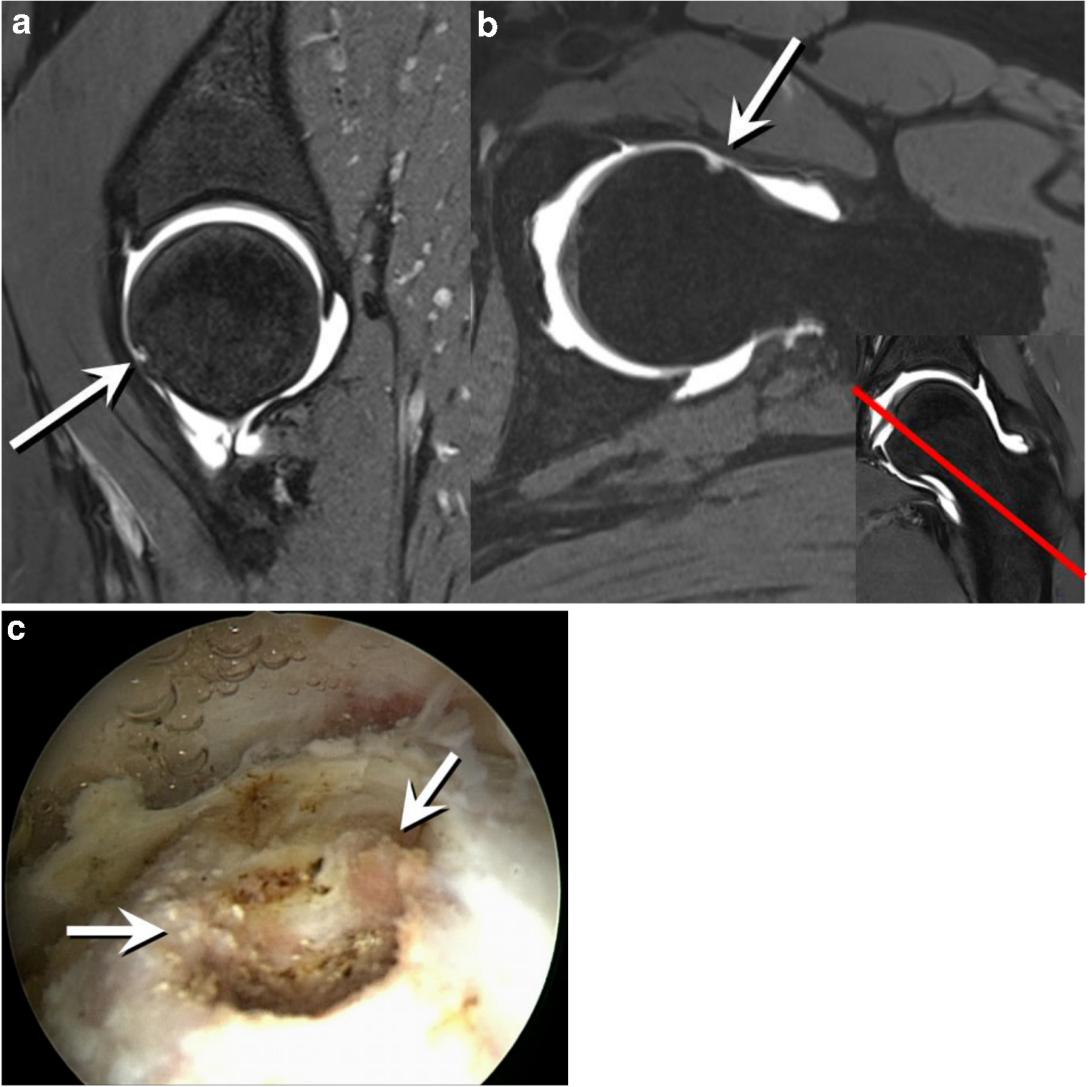
**a** Preoperative sagittal 3D double-echo steady-state MR arthrography image (repetition time msec/echo time msec 25/9) of the left hip of a 34-year-old woman with mixed-type FAI. Crater-like defect at the anteroinferior femoral head (arrow) corresponding to a type II FHD. **b** Transverse oblique three-dimensional water-excitation true fast imaging with steady-state precession MR arthrography image (repetition time msec/echo time msec, 12/6). Type II FHD at the anteroinferior femoral head (arrow). Insert in **b** Coronal image with a red reference line indicating the location of the transverse oblique image, confirming the location of the FHD in an anteroinferior location. **c** Arthroscopic intraoperative appearance of the FHD (arrow) of the same patient. The patient was arthroscopically treated for FAI by resection of the cam deformity (osteochondroplasty) as well as anterolateral remodeling of the damaged labrum (not depicted here)

Two of the patients with FAI included in the study population were scheduled for arthroscopic osteochondroplasty during this study. Arthroscopic images of the FHD were acquired during surgery. The following is a correlation of preoperative radiographic and MRI finding with the arthroscopic appearance of the FHD as captured by the hip surgeon during different stages of the procedure (Figs. 7 and 8).

## Discussion

Osseous defects of the anteroinferior femoral head have, though being a common finding, not been thoroughly examined so far. We have found only two reports in the literature [4, 17] which conclude that these lesions were a simple variant of the widely recognized herniation pit [3]. First described in 1981, the herniation pit was defined as a subcortical cystic lesion at the anterosuperior quadrant of the femoral head [2] caused by invagination of synovia into the bone through erosions or perforations. At arthroscopy, the herniation pit was described as having a pit-like or crater-like appearance [18]. After arthroscopic unroofing of the cystic lesion, there appeared a sclerotic bone base filled with gelatinous to fibrous-like material [18].

The location of the herniation pit has been identified as the anterosuperior quadrant of the femoral head [2, 18], the area that is thought to be the epicenter of abnormal mechanical abutment of proximal femur and the acetabular rim caused by femoroacetabular impingement (FAI). The surface of the femoral neck has historically been termed the “reaction area” [19]. Histologically, its surface area has been shown to display reactive changes with dense collagenous tissue covering neocartilage and an underlying layer of reactive new bone formation [2].

Early studies regarded herniation pits as normal variants with no clinical significance [3].

Later studies reported a correlation of herniation pits with femoroacetabular impingement (FAI) [20–26]. However, this association was later disputed as several other study groups found no correlation of herniation pits with FAI [1, 27–30].

The location of the femoral head defect (FHD) at the anteroinferior aspect of the femoral head differs from the more anterosuperior location of the extensively described herniation pit. Contrary to the mere two studies mentioning defects at the inferior femoral head [4, 17], we propose that the FHD is not simply a variant of the herniation pit, but rather its own entity, with both different location and morphology.

In our study population, the FHD was seen at the anteroinferior location of the femoral head: the overwhelming majority of FHD was located at the 8 o’clock position on a superimposed clockface. This differs from the herniation pit which is usually located in the anterosuperior quadrant of the hip joint and is often associated with cam-type deformities of the proximal femur.

The FHD was on average smaller (3.4 mm) than the herniation pit (7 mm) [18].

Abnormal contact of the femoral head-neck junction and the acetabulum due to FAI or acetabular overcoverage [13] seems not to have played a role in the development of FHD. Patients with and without FHD showed no significant difference in the number of individuals with pincer morphology, cam morphology, and abnormal LCE angles. Neither was there a correlation between the presence of an FHD and hip deformities with pathological femoral neck-shaft angles in the form of coxa vara or coxa valga.

Contrary to herniation pits, a correlation between FHD and FAI or any of its subtypes could not be established in this study. There was however a slight trend of FHD being associated with low femoral antetorsion. Reduced femoral antetorsion impairs the range of motion during internal rotation of the hip joint [31] causing increased mechanical impact between the femoral head and the anterior acetabular rim [14, 32, 33]. The FHD at the anteroinferior femoral head may, therefore, be associated with such repeated impaction in individuals with reduced femoral antetorsion.

Our study has limitations. In our study population, only patients with suspected hip pathology were included, and the study population did not include asymptomatic volunteers, so we cannot draw any conclusions about the frequency of FHD in the general population. Furthermore, a prospective study with arthroscopic confirmation of all FHD cases would be preferable to the retrospective design of our study. The absence of an association between FHD and FAI in our study population is not a definite proof that there is no such association; however, with a study sample of 970 MRI examinations and a *p* value of 0.890 that is much larger than the threshold for statistical significance, it seems unlikely that there is a correlation between these two entities.

Finally, our study setup did not allow evaluating small osteochondral defects of the femoral head due to trauma or early cartilage degeneration [34–36].

## Conclusion

The femoral head defect is a recurring finding in MRI examinations of the hip. FHD is not associated with FAI morphology. There is a non-significant trend towards lower femoral antetorsion in patients with FHD pointing to mechanical impaction as a possible cause for its development.
